# The functional significance of the skeletal muscle clock: lessons from *Bmal1* knockout models

**DOI:** 10.1186/s13395-016-0107-5

**Published:** 2016-10-13

**Authors:** Stefano Schiaffino, Bert Blaauw, Kenneth A. Dyar

**Affiliations:** 1 Venetian Institute of Molecular Medicine (VIMM), Via Orus 2, 35129 Padova, Italy; 2 Department of Biomedical Sciences, University of Padova, Padova, Italy; 3 Molecular Endocrinology, Institute for Diabetes and Obesity, Helmholtz Zentrum München, Munich, Germany

**Keywords:** Skeletal muscle, Circadian rhythms, Muscle clock, *Bmal1* knockout, Muscle denervation, Glucose uptake, Glucose metabolism

## Abstract

The circadian oscillations of muscle genes are controlled either directly by the intrinsic muscle clock or by extrinsic factors, such as feeding, hormonal signals, or neural influences, which are in turn regulated by the central pacemaker, the suprachiasmatic nucleus of the hypothalamus. A unique feature of circadian rhythms in skeletal muscle is motor neuron-dependent contractile activity, which can affect the oscillation of a number of muscle genes independently of the muscle clock. The role of the intrinsic muscle clock has been investigated using different *Bmal1* knockout (KO) models. A comparative analysis of these models reveals that the dramatic muscle wasting and premature aging caused by global conventional KO are not present in muscle-specific *Bmal1* KO or in global *Bmal1* KO induced in the adult, therefore must reflect the loss of *Bmal1* function during development in non-muscle tissues. On the other hand, muscle-specific *Bmal1* knockout causes impaired muscle glucose uptake and metabolism, supporting a major role of the muscle clock in anticipating the sleep-to-wake transition, when glucose becomes the predominant fuel for the skeletal muscle.

## Background

**Fig. 1:**
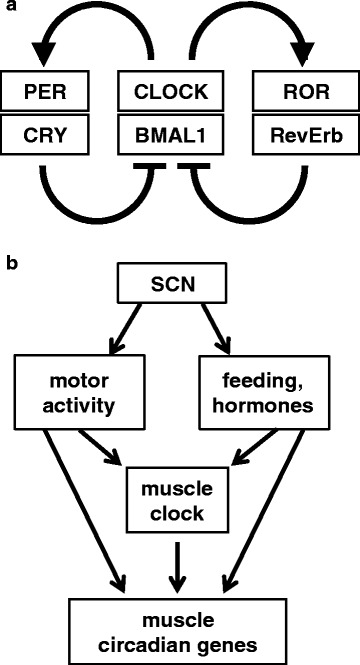
Core clock mechanism and control of circadian muscle genes by intrinsic and extrinsic pathways. **a** Simplified scheme of the core clock mechanism. The clock consists of a stimulatory loop, with the CLOCK-BMAL1 heterodimer stimulating the transcription of *Per* and *Cry* genes, and an inhibitory feedback loop with the PER-CRY heterodimer translocating to the nucleus and repressing the transcription of the *Clock* and *Bmal1* genes. An additional loop involves the ROR and RevErb factors. The different isoforms of the clock genes, including the genes coding for PER1 and PER2, CRY1 and CRY2, RORα and RORβ, and RevErbα and RevErbβ, are not indicated in the scheme. **b** The scheme illustrates how the master clock in the suprachiasmatic nuclei (SCN) of the hypothalamus controls motor activity and other systemic circadian rhythms (including feeding, hormone release, and body temperature), which in turn modulate the circadian rhythm of the muscle clock or directly dictate the oscillation of other muscle circadian genes. Modified from [7]

All tissues of the body display circadian oscillations in gene expression involving both the core clock genes, which are common to all tissues, and a large number of other genes, most of which are tissue-specific [1]. The core clock system consists of a transcriptional/translational feedback loop whereby a complex of two basic helix-loop-helix-PAS domain-containing transcription factors, BMAL1 and CLOCK, induces the expression of *Per* and *Cry* genes, whose products repress the transcription of *Bmal1* and *Clock*, thus inhibiting their own transcription (Fig. 1a) [2]. *Bmal1* expression is controlled by additional factors, RORs and REV-ERBs, whose transcription is also regulated by the BMAL1-CLOCK complex, and is further modulated by post-transcriptional changes, such as casein kinase (CK)2-mediated phosphorylation [3]. Most core clock genes show functional redundancy due to the presence of variants, such as *Per1* and *Per2*, or *Cry1* and *Cry2* genes, so that double knockouts are required to disrupt clock function; NPAS2 can substitute for CLOCK as a partner for BMAL1, at least in some tissues [2, 4]. In contrast, BMAL1 is a non-redundant clock component, and its ablation has been extensively used to determine the function of the muscle clock. The circadian oscillations of most cellular genes are controlled either directly by the intrinsic clock or by extrinsic factors, such as feeding, hormonal signals, or neural influences, which are regulated by the central pacemaker, the suprachiasmatic nucleus (SCN) of the hypothalamus. Here, we briefly review the regulation of circadian rhythms in the skeletal muscle, focusing on the role of the intrinsic muscle clock and critically considering the effects of different *Bmal1* knockout (KO) models. These studies reveal that the intrinsic muscle clock is dispensable for muscle growth and does not affect aging and life span, in contrast to previous suggestions based on global *Bmal1* KO. On the other hand, the muscle clock has a crucial function in muscle metabolism, by anticipating the changes in glucose uptake and oxidation at the sleep-to-wake transition.

## Extrinsic control of circadian rhythms in the skeletal muscle: the role of motor activity

Peripheral oscillators, including the muscle clock, are synchronized by the SCN through a variety of signals, including daily variations in body temperature, humoral factors, and the autonomic nervous system (reviewed in [2]). Oscillations of tissue-specific circadian genes are controlled either directly by the intrinsic peripheral clocks or indirectly by extrinsic factors (Fig. 1b). For example, inducible liver-specific repression of *Bmal1* transcription abrogates the oscillation of most circadian liver genes, showing that they are directly controlled by the hepatocyte clock; however, a number of other genes continue oscillating even in the absence of a functional liver clock, showing that they are controlled by extrinsic factors [5]. Feeding has a dominant role in setting the phase of peripheral oscillators, as temporal feeding restriction, induced by offering food only during the light phase, radically changes the phase of both core clock genes and other circadian genes in peripheral tissues of mice [6], including the skeletal muscle [7]. Plasma glucocorticoid and body temperature rhythms are also involved in the synchronization of peripheral clocks [2]. Another potential extrinsic circadian signal, which is unique to skeletal muscle, is motor neuron-dependent contractile activity.

Locomotor activity has traditionally been used both in mice and in flies as a readout of the circadian timing system. One may wonder whether motor neuron activity regulates the intrinsic muscle clock and/or other muscle cycling genes. Indeed, a phase distribution analysis of the circadian muscle transcriptome revealed that the largest cluster of rhythmic genes is found at the midpoint of the active phase [8]. Exercise was found to affect both the amplitude and the phase of the circadian clock in the skeletal muscle (reviewed in [9, 10]). However, interpretation of these results is complicated by the fact that exercise causes systemic effects, such as hormonal changes and increased body temperature, which are known to affect the peripheral oscillators. One-leg exercise in humans allowed for direct comparisons between active and inactive legs in the same individuals, thus excluding the potential contribution of systemic effects of exercise: the expression of core clock genes and downstream targets was modified in the exercised but not in the non-exercised contralateral leg; however, only two time points were examined in this study [11].

**Fig. 2:**
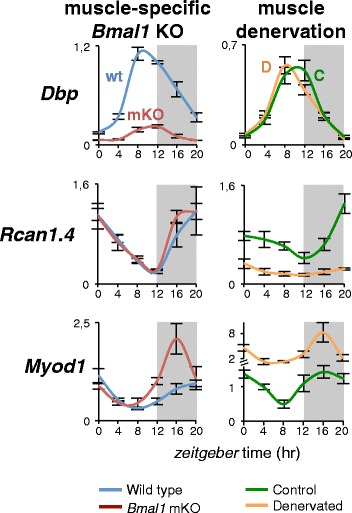
Changes in gene expression induced by muscle-specific *Bmal1* knockout (mKO), leading to disruption of the muscle clock, or denervation (*D*), leading to loss of motor activity. Transcript levels were monitored by qPCR every 4 h (0, lights on; 12, lights off). Three representative genes are illustrated. *Dbp*, a direct target of *Bmal1*, is strongly repressed by *Bmal1* KO but shows only a phase advance of around 4 h in denervated muscles without any change in oscillation amplitude. In contrast, *Rcan1.4*, a gene controlled by motor neuron activity via calcineurin-NFAT signaling, is essentially unchanged in *Bmal1* mKO muscles but is drastically downregulated by denervation. *Myod1*, coding for the myogenic regulatory factor MyoD, shows an atypical response, with circadian oscillation maintained with increased amplitude in both *Bmal1* mKO and denervated muscles. In denervation experiments, the muscles were removed 7 days after sciatic nerve section. The muscles examined were tibialis anterior for *Dbp* and *Myod1* and soleus for *Rcan1.4* (data from [27]; changes in *Myod1* are unpublished observations)

An alternative approach to define the role of nerve activity on circadian gene expression in the skeletal muscle is to compare fast-twitch, sporadically active muscles, composed by type 2 fibers, and slow-twitch, continuously active muscles, composed by type 1 fibers. Total daily activity, monitored by electromyography in rats, differs by more than 50 times between motor units composed of slow type 1 muscle fibers and motor units composed of fast-type 2B fibers [12]. The circadian expression pattern of the core clock genes was essentially the same in the two types of muscles, although most other circadian genes, including both clock-dependent and clock-independent genes, were specific for each muscle [7]. A more drastic experiment is to compare completely inactive denervated muscles with normally active contralateral muscles in the same animals. Core clock genes show small but significant changes in phase, amplitude, and/or expression level in the absence of motor nerve activity [7, 13]. For example, the circadian phase of *Bmal1* and its direct target, *Dbp*, is advanced by about 3–4 h in denervated muscles, but both amplitude and expression levels are unchanged (Fig. 2). In addition, many other clock-dependent and clock-independent muscle circadian genes show marked alterations in absolute expression level, phase, and amplitude. Importantly, the circadian oscillation of plasma glucocorticoids or the hepatic circadian expression of clock genes are not altered by nerve section, suggesting that systemic circadian rhythms are not affected in this experimental system [13].

The calcineurin-NFAT signaling pathway is involved in the nerve activity-dependent regulation of muscle fiber-type-specific gene programs (reviewed in [14]), and one member of the NFAT family, NFATc1, acts as a slow-type nerve activity sensor in vivo [15, 16], whereas other members of the NFAT family are also responsive to fast-type nerve activity and might thus contribute to modulate the fast fiber phenotype [17]. An NFATc1-GFP fusion protein, when electroporated in skeletal muscles in vivo shows a predominantly cytoplasmic localization in the fast tibialis anterior muscle but a nuclear localization in the slow soleus. A rapid nuclear translocation of NFATc1 can be induced by electrical stimulation of tibialis anterior with an impulse pattern typical of slow motor neurons, while denervation causes a rapid nuclear export of NFATc1 in soleus [16]. NFAT nuclear translocation and transcriptional activity has been recently analyzed during the day-night cycle [7]. NFATc1-GFP shows an accumulation in mouse soleus myonuclei during the dark phase with a peak at Zeitgeber (ZT)16, and a similar circadian oscillation of luciferase activity is seen after electroporation of an NFAT-luciferase construct but with a 4–8-h delay. The NFAT target gene, *Rcan1.4*, shows a similar circadian oscillation with a peak during the dark phase, which is drastically decreased in denervated muscle (Fig. 2). Interestingly, *Rcan1.4* circadian oscillation is unchanged in muscle-specific *Bmal1* mKO muscles, supporting the notion that *Rcan1.4* is a circadian muscle gene that is dependent on activity but independent of the core oscillator. It is possible that some of the changes induced by denervation may not reflect a direct effect of the loss of nerve activity but might be due to the transcriptional remodeling of gene expression that accompanies the denervation process. However, this possibility seems unlikely for NFAT target genes, as a drastic reduction of *Rcan1.4* gene expression during the dark phase is already detected at 12 h after nerve section, before any significant change in transcriptional programs has taken place in denervated muscles [7].

## The role of the intrinsic muscle clock: lessons from Bmal1 knockout models

The significance of the intrinsic muscle clock in muscle physiology has been addressed using genetic loss-of-function mouse models targeting the *Bmal1* gene. BMAL1 is a crucial component of the molecular clock, and the only one for which loss results in complete disruption of circadian rhythms [18]. Different mouse lines have been generated to inactivate the *Bmal1* gene in the skeletal muscles: as summarized in Table 1, these include both whole body and tissue-specific, constitutive, and inducible KO models.

**Table 1:** Global phenotypes of different *Bmal1* knockout models

	Whole body *Bmal1* KO			Muscle-specific *Bmal1* KO		
	Standard	Standard + rescue with *mBmal1* ^a^	Inducible	Standard	Inducible	
Circadian locomotor rhythm	No	No	No	Yes	Yes	Yes
Total activity level	↓	=	=	↑	=	=
Life span	↓	=	=	=	=	ND
Body weight	↓	=	=	=	=	ND
Muscle weight	↓	ND	ND^b^	↑	=	ND
Muscle fiber CSA	↓	ND	ND	↑	=	=
Muscle force (normalized)	↓	ND	ND	↓	=	↓
Muscle fiber-type profile (fast muscles)	ND	ND	ND	↓ 2X	=	↓ 2B
References	[18, 19, 26]	[32]	[30]	[27]	[27]	[31, 55]

*ND* not determined, = unchanged, ↓ decreased, ↑ increased, *CSA* cross-sectional area

^a^Mice with muscle-specific rescue of *Bmal1* null mice, obtained by crossing *Bmal1* global KO mice with *Bmal1* transgenic mice bearing a *Bmal1* DNA construct driven by the muscle-specific α-actin promoter

^b^Muscle weight was not determined but was likely unchanged because body weight and the weight of most organs, including fat deposits, were unchanged

Mice with whole body *Bmal1* KO, generated by standard methods leading to deletion of the gene in germinal cells, stop growing around 16 weeks of age, display progressive and dramatic muscle atrophy and decreased total activity level, and die between 26 and 52 weeks of age with signs of premature aging, including arthropathy, decreased hair growth, ocular abnormalities such as cataracts, and neurodegeneration with brain astrogliosis [18–20]. *Bmal1* KO also causes altered metabolism, including altered response to insulin [21, 22] and ectopic fat accumulation in the skeletal muscle [23]. Some of these changes could be due to increased oxidative stress, since the loss of *Bmal1* is known to cause accumulation of reactive oxygen species [19, 24] and antioxidant treatment was found to ameliorate symptoms of premature aging [25]. Muscle structure and function is altered in these mice even at early stages of postnatal development: at 12–14 weeks of age, muscle force is decreased, ultrastructural organization of thick and thin filament appears disrupted, and mitochondrial volume and respiratory function are decreased [26]. It was suggested that these changes are due to loss of function of the muscle-specific regulatory factor MyoD, because similar changes were found in *Myod1* null mice and *Myod1* was reported to be a target of BMAL1 and to lose its circadian oscillation in *Bmal1* KO mice [26]. However, this interpretation is in contrast with a subsequent study on a muscle-specific *Bmal1* KO model, obtained by crossing a mouse line bearing a floxed *Bmal1* with an *Mlc1f-Cre* line, bearing Cre recombinase driven by the myosin light chain 1 fast promoter [27]. These mice show drastic reduction of *Bmal1* transcripts in the skeletal muscle but not in the heart and other organs; however, they have normal life span and body weight with no obvious sign of premature aging. Muscle histology and ultrastructure are normal, and muscle weight is even increased with a slight decrease in normalized muscle force [27]. These findings suggest that the dramatic muscle atrophy found in whole body *Bmal1* KO mice cannot result from a disrupted muscle clock or from loss of cell-autonomous function of *Bmal1* in muscle fibers (see also [28]). In addition, *Myod1* gene expression is increased rather than decreased in these mice and maintains its circadian oscillation with a peak during the dark phase of the cycle (Fig. 2). A similar effect, with an even greater upregulation of *Myod1*, is seen after denervation. Based on these results, it seems unlikely that MyoD can mediate the effect of BMAL1 function on the skeletal muscle, as previously suggested [26]. On the other hand, *Myod1* gene expression is apparently controlled by feeding, as *Myod1* transcripts are strongly downregulated by fasting, under conditions when *Bmal1* transcripts maintain their normal levels and circadian pattern of expression [29].

Further insight into the function of *Bmal1* in muscle fibers was obtained by inducible *Bmal1* KO models. A ubiquitously inducible KO model was generated by crossing a floxed *Bmal1* line with a tamoxifen-inducible universal Cre line [30]. Tamoxifen treatment was started in 3-month-old mice leading to *Bmal1* inactivation in all tissues at an adult stage, with the skeletal muscles showing a 99 % reduction of *Bmal1* mRNA levels at Zeitgeber time 0 (ZT0, lights on), when *Bmal1* expression is high. These mice showed no significant difference in life span or body and organ weight when compared to control, suggesting that the dramatic phenotype seen in conventional *Bmal1* KO mice results from BMAL1 function during development. Hair growth is normal, and there is no sign of age-dependent arthropathy or calcification, although brain astrogliosis and ocular abnormalities, similar to those observed with prenatal *Bmal1* KO [20], were also evident after postnatal *Bmal1* depletion [30]. No difference was seen in glucose tolerance test (GTT) and insulin tolerance test (ITT) between KO and control mice. These results indicate that most phenotypes in conventional *Bmal1* KO mice, previously attributed to disruption of circadian rhythms, reflect the loss of properties of BMAL1 during early development and are probably independent of its role in the clock (see below). However, BMAL1 appears to have a direct function in the eye and central nervous system irrespective of developmental issues and probably due to increased oxidative stress [30]. Muscle-specific inducible models were generated by crossing a floxed *Bmal1* line with a tamoxifen-inducible Cre driven by the human α-actin promoter, thus inducing *Bmal1* inactivation exclusively in the skeletal muscle at an adult stage [27]. These mice have an essentially normal phenotype with respect to life span, body weight, and muscle mass (Table 1); however, they show altered glucose metabolism (see below). Muscle force and the proportion of type 2B fibers were decreased in these mice, whereas fiber size was unchanged even at 12 months of age and centrally nucleated fibers were not detected [31].

Taken together, these studies suggest that the dramatic phenotype observed in the global *Bmal1* KO, characterized by premature aging and death, with reduced body weight and muscle wasting, reflects the loss of *Bmal1* function during development in non-muscle tissues. Two KO models support this conclusion. First, muscle-specific *Bmal1* KO, leading to *Bmal1* deletion since early developmental stages selectively in the skeletal muscle, does not induce significant changes with the exception of altered muscle metabolism [27], pointing to a major effect of *Bmal1* in non-muscle tissues in the pathogenesis of sarcopenia, premature aging, and reduced life span. Second, these changes are also absent when *Bmal1* KO is induced ubiquitously at an adult stage, therefore must reflect the loss of *Bmal1* during development [30].

**Fig. 3:**
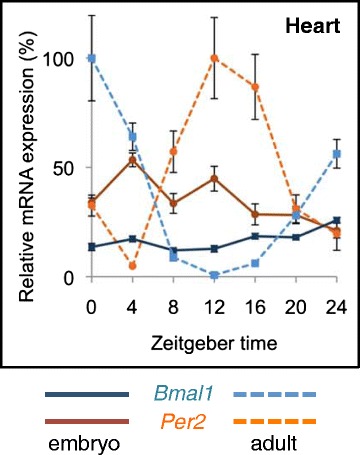
*Bmal1* and *Per2* transcripts do not oscillate in embryonic tissues. Twenty-four-hour expression profiles of *Per2* and *Bmal1* mRNA in embryonic (E18-E19) and adult mouse heart, as determined by qPCR. Identical results were seen in the liver and kidney, while the skeletal muscle was not analyzed in this study. Note that the embryonic heart shows little circadian variation in *Per2* and *Bmal1* expression, in contrast with the robust changes seen in the adult tissue (modified from [39])

However, another experimental model seems to contradict this interpretation. Mice with muscle-specific rescue of *Bmal1* null mice, obtained by crossing *Bmal1* global KO mice with *Bmal1* transgenic mice bearing a *Bmal1* DNA construct driven by the muscle-specific human α-actin promoter, show normal body weight and total activity level, as well as longer life span compared to *Bmal1* null mice [32]. Although incomplete information was provided on some critical aspects (life span profile, muscle weight), which does not allow a thorough evaluation of this model, some considerations could be put forward to explain this surprising result. A number of studies in *Drosophila* have revealed that the skeletal muscle can affect the global body phenotype (reviewed in [33, 34]). This conclusion is supported by studies in mammals, for example, reduced body size, but essentially normal skeletal muscle phenotype, is observed when myogenin, a muscle-specific transcription factor, is deleted at birth using an inducible KO model [35]. Some effects of the skeletal muscle on the whole organism can be mediated by the release of secreted factors from muscle cells. Hundreds of muscle genes, potentially coding for secreted proteins, are expressed in skeletal muscle [36], and specific myokines have been identified (reviewed in [37]). Myokines can transmit signals from the muscles to adipose tissue or other organs and, in this way, affect global aspects of the phenotype (see [33, 34]). Interestingly, the secretion of several myokines is strongly downregulated by siRNA targeting *Clock* in skeletal myotubes, showing that the muscle clock is involved in the regulation of basal myokine secretion [38]. One wonders whether the muscle *Bmal1*-dependent rescue of the phenotype of *Bmal1* null mice is mediated by a myokine release mechanism. *Bmal1*, like other core clock genes, is expressed since early embryonic stages in both the SCN and peripheral tissues but does not oscillate during embryogenesis (Fig. 3), clock rhythm being established during the first week after birth [39]. The role of *Bmal1* in the embryo is not known, but one possibility is that this gene might have specific non-clock-related functions, for which loss might account for premature aging [30]. Indeed, a recent study indicates that BMAL1 stimulates translation independently of its transcriptional activity. The ribosomal S6 protein kinase 1 (S6K1), an important mTOR-dependent regulator of translation, rhythmically phosphorylates BMAL1, which in turn interacts with the translational machinery, promoting protein synthesis [40].

## Role of the muscle clock in glucose uptake and metabolism

**Fig. 4:**
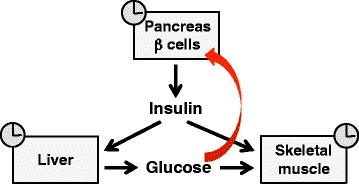
The scheme illustrates the role of peripheral clocks in the control of muscle glucose metabolism, as determined using tissue-specific *Bmal1* knockout models. The liver clock controls glucose output during the fasting/inactive phase, as shown by the finding that liver-specific *Bmal1* KO causes hypoglycemia during this phase [42]. The pancreas β cell clock controls insulin secretion, as β cell-specific *Bmal1* KO causes hyperglycemia [43–45]. The muscle clock promotes glucose uptake and metabolism at awakening, as skeletal muscle-specific *Bmal1* KO causes impaired insulin-dependent glucose uptake and glucose oxidation in skeletal muscle fibers [27]

Glucose metabolism is altered by disruption of circadian rhythms induced by SCN lesion or global inactivation of clock genes (see [41]). However, both types of interventions profoundly affect behavior, including locomotor activity and feeding rhythms, and may thus indirectly alter metabolism. Tissue-specific *Bmal1* KO models allow to identify the direct role of local clock mechanisms in glucose metabolism in the presence of normal locomotor activity and feeding rhythms. These models have revealed the crucial contribution of peripheral clocks in the pancreas, liver, and skeletal muscle on glucose metabolism (Fig. 4). Liver-specific ablation of *Bmal1* causes hypoglycemia during the fasting phase, when plasma glucose is controlled by the liver through glycogenolysis and gluconeogenesis, due to loss of rhythmic expression of hepatic glucose regulatory genes, responsible for hepatic glucose production and export [42]. Selective inactivation of *Bmal1* in the pancreas or in the β cells causes hyperglycemia due to impaired insulin secretion [43–45], and isolated pancreatic islets from adult mice display circadian oscillation in glucose-stimulated insulin secretion which is abrogated by *Bmal1* ablation [46]. Transcriptome analyses showed that a large number of genes involved in insulin secretion display circadian changes in expression and chromatin immunoprecipitation revealed that CLOCK and BMAL1 control cycling genes in β cells by binding at distal regulatory elements. Severe glucose intolerance is induced in adult mice using a tamoxifen-inducible model of pancreas-specific *Bmal1* knockout [46]. On the other hand, glucose metabolism in adipose tissue is apparently unaffected by the local clock, as adipose tissue of mice with adipocyte-specific KO of *Bmal1* shows normal insulin sensitivity compared to control littermates despite the greater body adiposity [47].

**Fig. 5:**
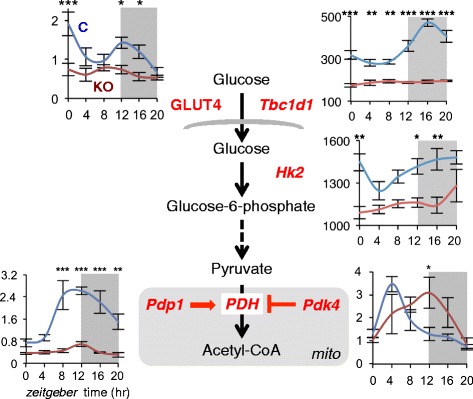
Simplified scheme of glucose uptake and metabolism in muscle cells, highlighting two crucial steps controlled by the intrinsic muscle clock: insulin-dependent glucose uptake and pyruvate conversion to acetyl-CoA. Insulin promotes glucose uptake by activating the kinase AKT that phosphorylates the Rab-GTPase-activating protein TBC1D1, thus promoting the translocation of GLUT4 to the plasma membrane. Pyruvate, upon entry into mitochondria (*mito*), is metabolized to acetyl-CoA by pyruvate dehydrogenase (PDH), whose activity is inhibited by the PDH kinase PDK4 and stimulated by the PDH phosphatase PDP1. The protein expression of GLUT4, and both mRNA and protein levels of TBC1D1, PDK4, and PDP1 vary across the day-night cycle (0, lights on; 12, lights off) and are drastically affected by *Bmal1* mKO. Under normal conditions, PDK4 has a peak of expression in the fasting phase (around ZT4), whereas PDP1 peaks around the transition from the fasting to the feeding/active phase (around ZT12). Note that PDK4 starts to decrease and PDP1 to increase during the fasting phase, before awakening, supporting the notion of the anticipatory role of the muscle clock, which prepares the muscles to the upcoming activity period. These circadian adaptations are completely disrupted by *Bmal1* mKO, with downregulation of PDP1 and a rightward shift in the peak of PDK4, leading to decrease in PDH activity at awakening (data from [27])

The skeletal muscle is the predominant site of insulin-stimulated glucose disposal in the postprandial state [48], and muscle insulin resistance is one of the earliest factors in the pathogenesis of the metabolic syndrome [49, 50]. Dyar et al. [27], using both constitutive and inducible muscle-specific *Bmal1* KO models, revealed that the muscle clock controls glucose uptake and metabolism. This conclusion was based on studies of gene expression at the transcript and protein level, integrated by enzymatic assays, glucose uptake studies, and circadian metabolomics analyses. Insulin-stimulated glucose uptake is impaired in the skeletal muscles from muscle-specific *Bmal1* KO mice, likely due to reduced transcript and protein levels of glucose transporter 4 (GLUT4), the insulin-dependent glucose transporter (Fig. 5). GLUT4 protein levels were reduced by 45 % across the diurnal cycle, a change similar to that seen in heterozygous GLUT4^+/−^ mice, which also show a reduced insulin-stimulated glucose uptake in the skeletal muscles [51]. Muscle-specific *Bmal1* KO mice are also characterized by reduced transcript and protein levels of TBC1D1, a Rab-GTPase involved in GLUT4 translocation to the plasma membrane. *Tbc1d1* KO mice likewise show a 50 % reduction in GLUT4 levels and impaired insulin-stimulated glucose uptake in the skeletal muscle [52]; therefore, it is likely that the loss of BMAL1 in the skeletal muscle causes decreased TBC1D1 levels which in turn leads to reduced GLUT4 and impaired insulin-stimulated glucose uptake. TBC1D1 has a circadian oscillation with a peak in the active/feeding phase but starts to increase already in the late fasting phase. In contrast, the expression of another Rab-GTPase involved in GLUT4 translocation, TBC1D4, shows no circadian oscillation in the skeletal muscle and is unaffected by *Bmal1* KO [27]. The phosphorylation of glucose to glucose-6-phosphate by hexokinase is also affected by the muscle clock, as suggested by the significantly reduced levels of hexokinase 2 (HK2) after *Bmal1* inactivation. Pyruvate dehydrogenase (PDH) activity is reduced in the skeletal muscles due to altered expression of circadian genes *Pdk4* and *Pdp1*, coding for PDH kinase and phosphatase, respectively. Under normal conditions, PDH kinase 4 (PDK4), which inhibits PDH activity, shows a peak of expression during the fasting phase (around ZT4), whereas PDH phosphatase 1 (PDP1), which stimulates PDH activity, peaks around the transition from the fasting to the feeding phase (around ZT12). Interestingly, PDK4 starts to decrease whereas PDP1 begins to increase during the fasting phase, before awakening, supporting the notion of the anticipatory role of the muscle clock, which prepares the muscles for the upcoming activity period. Accordingly, muscle clock disruption leads to PDH inhibition, thus uncoupling glycolysis from glucose oxidation, and contributing to the diversion of glycolytic intermediates to alternative metabolic pathways, as revealed by metabolomics analysis [27].

The tissue-specific role of BMAL1 in regulating insulin sensitivity in muscle cells is supported by studies on cultured C2C12 myotubes [53]. In these cells, insulin sensitivity is reduced by knockdown of *Clock* or *Bmal1*, while the insulin resistance induced by palmitate is improved by *Clock* and *Bmal1* overexpression [53]. These effects appear to be mediated by SIRT1, which is a target of CLOCK and BMAL1, as shown by the finding that (i) *Sirt1* knockdown blocks the improvement induced by *Clock* and *Bmal1* overexpression on the palmitate-dependent insulin resistance and (ii) *Sirt1* overexpression ameliorates insulin resistance induced by knockdown of *Clock* or *Bmal1*.

## Conclusions

The comparative analysis summarized in Table 1 shows that the muscle phenotype is variably affected in different *Bmal1* KO models. In particular, the dramatic muscle wasting and premature aging found in the global conventional KO are not present in muscle-specific *Bmal1* KO or in global KO induced in the adult, thus must reflect the loss of *Bmal1* function during development and in non-muscle tissues. These findings indicate that the intrinsic muscle clock is dispensable for muscle growth and that its inactivation does not cause premature aging and reduced life span. The fact that core clock genes are not oscillating in embryonic tissues points to possible non-clock functions of *Bmal1* during development, such as the recently identified BMAL1 role in the control of protein synthesis. On the other hand, *Bmal1* circadian oscillation in the skeletal muscle is involved in adult muscle metabolism. In particular, two models of muscle-specific inactivation of *Bmal1* suggest that the intrinsic muscle clock controls both glucose uptake and glucose metabolism in the skeletal muscle and support the conclusion that “a major physiological role of the muscle clock is to prepare the tissue for the transition from the rest/fasting phase to the active/feeding phase, when glucose becomes the predominant fuel for skeletal muscle” [27]. It will be important to confirm this conclusion with muscle-specific KO of other clock genes, e.g., double KO of *Per1* and *Per2*, or *Cry1* and *Cry2*, in order to establish unambiguously that the changes in muscle metabolism induced by *Bmal1* KO result from the disruption of the muscle clock and not from specific functions of *Bmal1*.

### Note added in proof

After submission of our manuscript we became aware of a recent study describing another muscle-specific *Bmal1* KO model obtained by crossing floxed *Bmal1* with MCK-Cre mice [54]. These mice have a normal life span, thus confirming the results of Dyar et al. [27], and show a denervation-induced increased in *Myod1* expression similar to that of wild type mice.

## Abbreviations

CK2: Casein kinase 2
GLUT4: Glucose transporter 4
GTT: Glucose tolerance test
HK2: Hexokinase 2
ITT: Insulin tolerance test
KO: Knockout
PDH: Pyruvate dehydrogenase
PDK4: PDH kinase 4
PDP1: PDH phosphatase 1
SCN: Suprachiasmatic nucleus
ZT: Zeitgeber
